# Aggressive Chemotherapy and Antepartum Management of Small-Cell Carcinoma of the Ovary of Hypercalcemic Type: A Novel Case Report with Ethical and Medical Decision-Making Consideration

**DOI:** 10.1055/a-2764-3698

**Published:** 2025-12-29

**Authors:** Hind N. Moussa, Dana Rector, Wesley Gherman, Eric Shuffle, Robert Fresch, Thomas Reid

**Affiliations:** 1 Division of Maternal Fetal Medicine, Department of Obstetrics and Gynecology, University of Cincinnati, Cincinnati, Ohio, United States; 2 Department of Maternal Fetal Medicine, Maternal Fetal Medicine, Kettering Health, Kettering, Ohio, United States; 3 Department of Obstetrics and Gynecology, University of Toledo and ProMedica Toledo Hospital, Toledo, Ohio, United States; 4 Department of Obstetrics and Gynecology, TriHealth, Cincinnati, Ohio, United States

**Keywords:** small-cell carcinoma of the ovary, hypercalcemic type, chemotherapy, biomedical ethics

## Abstract

**Background:**

Small-cell carcinoma of the ovary, hypercalcemic type (SCCOHT) is a rare, aggressive, and highly fatal gynecologic malignancy with few treatment guidelines offered in nonpregnant patients.

**Case Report:**

A 32-year-old woman presented for initial prenatal care at 9 weeks and her dating ultrasound was notable for a large complex adnexal mass. Magnetic resonance imaging performed 2 weeks later revealed accelerated interval growth with possible malignant etiology. She underwent surgical evaluation with salpingo-oophorectomy. Pathology noted signature features of SCCOHT. The patient desired expectant management and declined termination of pregnancy or chemotherapy. Four weeks later, she developed severe abdominal pain, nausea, and vomiting, and she was diagnosed with extensive metastatic disease. After multidisciplinary and multi-institutional counseling, she elected for continuation of her pregnancy and 6-agent chemotherapy including Vinblastine, Cisplatin, Cyclophosphamide, Bleomycin, Doxorubicin, Etoposide (VPCBAE) with close antepartum surveillance. The patient completed multiple rounds of chemotherapy and subsequently delivered via primary cesarean at 27 ^2/7^ weeks due to maternal sepsis and nonreassuring fetal status. Maternal and neonatal death occurred approximately 1-week postpartum.

**Conclusion:**

This is the first known case of SCCOHT in which the pregnancy was continued through an aggressive chemotherapy regimen.

## Introduction

Small-cell carcinoma of the ovary, hypercalcemic type (SCCOHT) is a rare, aggressive cancer that mainly occurs in adolescents and young women. First described by Dickersin et al in 1982, it represents less than 0.01% of all ovarian malignancies. 1 2 However, outcomes remain poor, with 5-year survival at 55, 40, and 29% for International Federation of Gynecology and Obstetrics (FIGO) Stages I, II, and III, respectively. 3 Multiple groups characterized SCCOHT by both germline and somatic deleterious mutations in SMARCA4, although studies show that SMARCA4 appears as the only recurrently mutated gene in SCCOHT. 4 5 6 Despite this, most recently, a study showed germline mutations in almost half of patients without any family history of SCCOHT. 7

Until recently, treatment recommendations lacked consensus. However, in 2020, the International SCCOHT Consortium proposed surveillance and treatment options. 8 Treatment typically involves nonconservative cytoreductive surgery and high-dose chemotherapy (HDC), with Vinblastine, Cisplatin, Bleomycin, Cyclophosphamide, Doxorubicin, Etoposide (VPABCE) being the most researched regimen. This combination decreases rates of recurrence compared with cisplatin/etoposide or no adjuvant chemotherapy. Major toxicities include myelosuppression, neutropenic fever, nausea/vomiting, and polyneuropathy. 9

As in our report, cases of SCCOHT in pregnancy are exceedingly rare, and there are limited data in the literature. Treatment of SCCOHT in pregnancy offers an ethical challenge involving considerations for both the mother and the fetus. Here, we report a case of SCCOHT in pregnancy where the patient elected for continuation of the pregnancy along with aggressive chemotherapy.

## Case Report

**Fig. 1:**
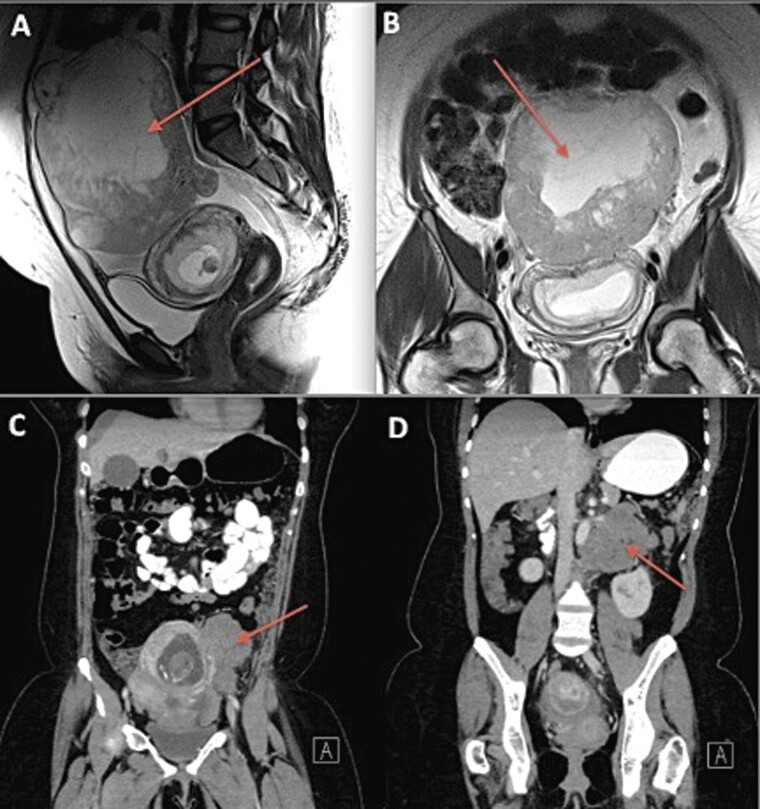
( **A** ) MRI sagittal view of tumor preoperative. ( **B** ) MRI coronal view of tumor preoperative. ( **C** ) CT coronal view pelvic metastasis 4 weeks postoperative. ( **D** ) CT coronal view abdominal metastasis 4 weeks postoperative. MRI, magnetic resonance imaging.

**Fig. 2:**
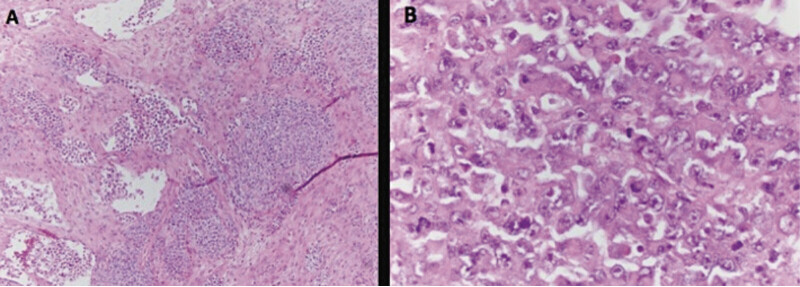
( **A** ) Left ovary, Small-cell carcinoma hypercalcemic type, large-cell variant. Diffuse pattern showing tumor cells with open nuclei, prominent nucleoli and moderate nucleated cytoplasmic ratio. Brisk mitotic activity is present along with patchy necrosis. ( **B** ) Typical morphology including the presence of a component of the so-called large-cell variant.

**Fig. 3:**
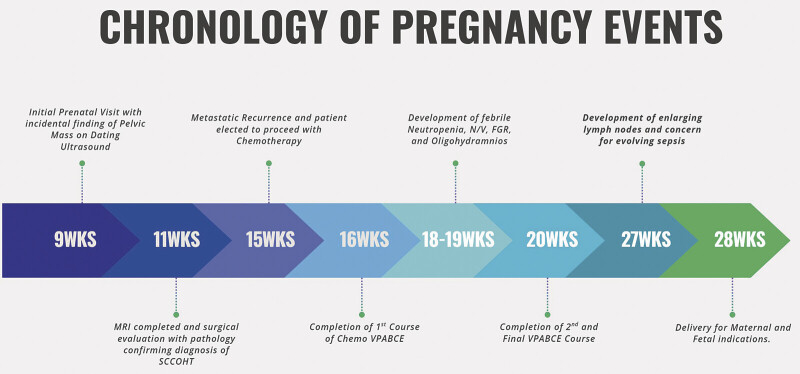
Chronology of pregnancy events.

In this evaluation, a 32-year-old gravida 1 para 0 (G1P0) female presented to her obstetrician at 9 weeks' gestation for initial prenatal care and dating ultrasound, which showed a large pelvic mass, measuring approximately 11 cm with solid and cystic components. A magnetic resonance imaging performed approximately 2 weeks later revealed the same mass, measuring 15 cm in greatest diameter along with a small amount of ascites ( Fig. 1 ). She subsequently underwent multidisciplinary counseling and active management including surgical evaluation with exploratory laparotomy and subsequent removal of the ovarian mass along with the adjacent fallopian tube and evacuation of ascites. The pathology slides were sent for a second opinion to leading experts in the field, where they described signature features consistent with SCCOHT along with the presence of a large-cell variant ( Fig. 2 ). At diagnosis, the patient declined the recommended cytoreductive surgery and pregnancy termination. Multi-institutional and multidisciplinary counseling reinforced the patient's poor prognosis, who decided to continue with expectant management for fetal benefit to support the patient's informed decision. At 15 ^5/7^ weeks, the patient developed severe abdominal pain, nausea, and vomiting with repeat imaging showing omental, peritoneal, and para-aortic metastasis. At this point, the gynecology oncology team initiated another multi-institution care team involving regional experts to revisit the patient's decision-making regarding the advancing disease. With more information, the patient decided to proceed with treatment despite being given the recommendation to terminate the pregnancy. The chemotherapy regimen, VPABCE, began immediately alongside close antepartum surveillance. Prior to the initiation of multiagent chemotherapy, a detailed discussion of potential maternal and fetal risks was had with the patient. Reported maternal toxicities include myelosuppression, infection, nausea, and organ-specific effects such as nephrotoxicity and cardiotoxicity. Fetal risks vary by timing of exposure, with first-trimester administration associated with higher risks of teratogenicity, whereas second- and third-trimester exposure may contribute to growth restriction or preterm delivery ( Table 1 ). 10 11 12 13 14 15 16 17 18 After completing a regimen of VPABCE, the patient presented at 18 weeks with febrile neutropenia and intractable nausea and vomiting. Despite prophylactic cefepime, cultures showed no growth. At 19 weeks, fetal ultrasound demonstrated oligohydramnios with severe fetal growth restriction despite sensations of normal fetal movements. She completed her second course of VPABCE a week later. At almost 27 weeks, an enlarging left supraclavicular lymph node caused hoarseness without airway compromise. A few days later, the fetus delivered via primary cesarean section secondary to maternal sepsis, nonreassuring fetal status, and growth restriction ( Fig. 3 ). Three days after delivery, the patient became hypotensive, anuric, and febrile with abdominal distention. After confirming abdominal compartment syndrome unrelieved by paracentesis and agreeing upon a code status change to Do Not Resuscitate—Comfort Care Arrest, she passed later that evening. Of note, the newborn had severe fetal growth restriction and complications at the time of delivery included: inability to pass nasogastric/orogastric tube, esophageal perforation, and respiratory failure unresponsive to multiple interventions. There were no suspected anomalies impacting resuscitation efforts. The newborn ultimately passed at 4 days of life.

**Table 1:** Chemotherapy agents and associated maternal and fetal side effects

Chemotherapy agents	Maternal risks	Fetal/neonatal risks	References
Vinblastine	Myelosuppression, risk of sepsis, peripheral neuropathy, gastrointestinal toxicity	Transient neonatal myelosuppression and cytopenias, IUGR and preterm birth,1 case of plagiocephaly and syndactyly, but otherwise no consistent pattern of malformations with 2nd–3rd trimester use	NIH, 10 AYA, 11 Brenner 2012 12
Cisplatin	Nephrotoxicity, myelosuppression, risk of sepsis, peripheral neuropathy, ototoxicity, nausea, and vomiting	Oligohydramnios, IUGR, and preterm birth. Cases of neonatal acute respiratory distress syndrome, transient cytopenias, and hearing loss, 1 case of cerebral ventriculomegaly and cerebral atrophy	NIH, 13 AYA 11
Cyclophosphamide	Myelosuppression, risk of sepsis, hemorrhagic cystitis, gastrointestinal toxicity, nausea and vomiting, alopecia, hepatotoxicity, cardiotoxicity, pulmonary toxicity	Teratogenic in 1st trimester (craniofacial, limb, and organ malformations), miscarriage, IUGR, 2nd–3rd trimester linked to IUGR and cytopenias	NIH, 14 AYA, 11 Brenner 2012 12
Bleomycin	Pulmonary toxicity (pneumonitis, fibrosis), skin toxicity	Teratogenic in 1st trimester (skeletal malformations, hydroureter), 2nd–3rd trimester side effects include: IUGR, preterm birth, 1 case of plagiocephaly and syndactyly	NIH, 15 AYA, 11 Brenner 2012 12
Doxorubicin	Cardiotoxicity, myelosuppression, risk of sepsis, gastrointestinal toxicity, secondary malignancies (acute myelogenous leukemia and myelodysplastic syndrome)	Teratogenic in 1st trimester (holoprosencephaly, esophageal and intestinal atresia, cardiovascular anomalies), risk of intrauterine fetal death and spontaneous abortion, 2nd–3rd trimester effects include: IUGR, preterm birth	NIH, 16 AYA, 11 Brenner 2012, 12 Germann 2004 17
Etoposide	Myelosuppression, risk of sepsis, secondary leukemias, hypersensitivity reactions	Teratogenic in 1st trimester in animal studies (exencephaly, encephalocele, anophthalmia) risk of miscarriage, fetal death, 2nd–3rd trimester effects include IUGR, preterm birth, and transient neonatal cytopenias	NIH, 18 AYA, 11 Brenner 2012 12

Abbreviations: AYA, Adolescent and Young Adult (Oncology); IUGR, intrauterine growth restriction; NIH, National Institutes of Health.

Notes: This is not a comprehensive list of side effects, but rather a summary of some of the most common or severe side effects given the limited data available in human and animal studies. Additionally, many of these are used in multiagent regimens so individual side effects are sometimes difficult to ascertain.

## Discussion

This challenging case involved highly complex clinical and ethical decision-making. A highly motivated patient chose to continue her pregnancy while on chemotherapy, despite the uncertain outcome involved with treating SCCOHT. A number of sequelae complicated the gestational period including open surgical intervention, ovarian intra-abdominal metastases, multiple rounds of HDC, febrile neutropenia, multiple episodes of nausea/vomiting, fetal growth restriction, oligohydramnios, and an emergency cesarean section secondary to nonreassuring fetal heart tracing. All resulted in the unfortunate demise of both patient and her child several days later despite a good state of health before this cascade of events and no significant contributing familial oncologic history. The confirmatory pathological reading from a long-standing expert on the topic of SCCOHT commented that the large-cell variant made the already poor prognosis of the cancer diagnosis even worse. This case highlighted the importance of a multidisciplinary approach to care, especially in complex patients with poor prognoses.

Limited reports in the literature note SCCOHT in pregnancy. A case reported in 2009 by McCormick et al described a patient diagnosed with a Stage IIB SCCOHT at 15 weeks' gestation who was treated with immediate cytoreductive surgery and intrapartum conservative chemotherapy with etoposide and cisplatin until delivery at 35 weeks due to eclampsia. She underwent a 6-week postpartum exploratory laparotomy with optimal cytoreductive surgery including hysterectomy. However, she was lost to follow-up and later discovered to have passed away 10 months after initial diagnosis due to unspecified disease recurrence. 19 Another case by Tewari et al described a patient diagnosed with SCCOHT at 13 weeks' gestation who underwent immediate surgery including total abdominal hysterectomy, bilateral salpingo-oophorectomy, tumor debulking, and aggressive VPCBAE chemotherapy; she remained alive 5.5 years after diagnosis without evidence of recurrence. 20 Finally, a case report by Phoolcharoen et al followed a 26-year-old who presented to her gynecologist with abdominal pain, distension, and emesis. Further evaluation revealed a complex solid pelvic mass, which was surgically removed along with the ipsilateral fallopian tube. Biopsy proved to be SCCOHT; 6 cycles of VPBCAE chemotherapy proved successful and the patient spontaneously became pregnant 6 years later. 21 In view of the morbidity with this type of malignancy, publication bias makes the outcome of other published cases favorable.

Given the young age at diagnosis, many reproductive-age women elect to preserve fertility if given the option. Cancer diagnosed in pregnancy is a relatively rare event, occurring in approximately 0.01 to 0.03% of all pregnancies. 22 There are limited data on fertility and pregnancy outcomes following SCCOHT treatment beyond those mentioned above. Fertility preservation and pregnancy termination present difficult ethical situations. A survey study conducted by Han et al involving gynecologic oncologists and general obstetrician gynecologists in 14 European countries found that 44% of practitioners would offer termination as the primary treatment option if malignancy is diagnosed in the first trimester of pregnancy. 23 In addition, for those patients already pregnant, balancing therapy plans and fetal welfare becomes even more critical. Chemotherapy, immunotherapy, radiotherapy, and cytoreductive surgical options all impact maternal and fetal well-being.

The “principlism” approach to biomedical ethics uses a framework of four universal and basic ethical principles; respect for autonomy, nonmaleficence, beneficence, and justice ( Table 2 ). 24 A systematic review considers patient autonomy and its relational aspects an integral part of future clinical practice guidelines. In this way, the patient has the right to make her own decision about whether or not to continue with her pregnancy while also considering the impact that oncologic treatment could have on the fetus. Also, other principles to consider include a balanced approach to maternal and fetal beneficence, protection of the vulnerable, and justice for resource allocation of cancer treatment for all pregnant women. 25 Several other pragmatic ethical frameworks that offer guidance in these situations include case-based analysis, ethics of care, and feminist theory; however, principlism tends to be the most popular. As mentioned within the principlism framework, the fetus has no claim to patient status that is independent of the pregnant woman's autonomy. However, as discussed in Alpuim Costa et al, emphasizing the differing interests of mother and fetus minimizes the more important shared goals. 26 In our case, all interests aligned; the treatment of the mother's cancer with chemotherapy while maintaining the health and safety of the fetus, balancing the beneficence. The pregnancy was continued and neonatal death was related to prematurity care complications. In summary, the best ethical decision follows a multistep process including an unbiased presentation of the available treatment options.

**Table 2:** Ethical principles of this case report

Ethical principle	Definition	Application to this case	Key considerations
Autonomy	Respecting the patient's right to make informed decisions about her care	The patient was fully informed of prognosis and treatment options, including termination versus continuation of pregnancy. She autonomously chose to continue the pregnancy and pursue chemotherapy	Requires clear communication, full disclosure of risks/benefits, and respect for informed maternal choice even when prognosis is poor
Beneficence	Acting for the benefit of the patient and fetus; promoting well-being	The care team sought to balance treatment of maternal cancer with efforts to preserve fetal viability through multidisciplinary management	Joint goal of maternal and fetal benefit guided by the patient's values
Nonmaleficence	Obligation to avoid causing harm	Clinicians recognized that chemotherapy posed significant maternal and fetal risks but sought to minimize harm through close monitoring and evidence-based treatment	Includes preventing unnecessary interventions and reducing treatment-related morbidity
Justice	Fair distribution of health care resources and equitable treatment	The team ensured the patient received equal access to expert oncologic and obstetric care despite poor prognosis	Also encompasses fair consideration of maternal–fetal interests and allocation of intensive care resources
